# Controlling the simultaneous production of laccase and lignin peroxidase from *Streptomyces cinnamomensis *by medium formulation

**DOI:** 10.1186/1754-6834-5-15

**Published:** 2012-03-20

**Authors:** Debing Jing, Jinghua Wang

**Affiliations:** 1 College of Life Sciences, Capital Normal University, Beijing 100048, China; 2 Division of Food Biotechnology, Department of Food Sciences and Technology, BOKU University of Natural Resources and Applied Life Sciences, Vienna A-1190, Austria; 3 China Rural Technology Development Center, Beijing, 100045, China

## Abstract

**Background:**

Use of crude ligninase of bacterial origin is one of the most promising ways to improve the practical biodegradation of lignocellulosic biomass. However, lignin is composed of diverse monolignols with different abundance levels in different plant biomass and requires different proportions of ligninase to realize efficient degradation. To improve activity and reduce cost, the simultaneous submerged fermentation of laccase and lignin peroxidase (LiP) from a new bacterial strain, *Streptomyces cinnamomensis*, was studied by adopting formulation design, principal component analysis, regression analysis and unconstrained mathematical programming.

**Results:**

The activities of laccase and LiP from *S. cinnamomensis *cultured with the optimal medium formulations were improved to be five to eight folders of their initial activities, and the measured laccase:LiP activity ratios reached 0.1, 0.4 and 1.7 when cultured on medium with formulations designed to produce laccase:LiP complexes with theoretical laccase:LiP activity ratios of 0.05 to 0.1, 0.5 to 1 and 1.1 to 2.

**Conclusion:**

Both the laccase and LiP activities and also the activity ratio of laccase to LiP could be controlled by the medium formulation as designed. Using a crude laccase-LiP complex with a specially designed laccase:LiP activity ratio has the potential to improve the degradation of various plant lignins composed of diverse monolignols with different abundance levels.

## Background

Lignocellulose degradation is the central process for carbon recycling in land ecosystems [1]. As the key step in lignocellulose decay, lignin degradation, removal or modification is the rate-limiting step of carbon recycling [1,2], and also the central issue for industrial utilization of plant biomass (for example, biofuel production from abundant and renewable lignocellulosic material) [3,4]. Compared with the lignocellulosic biomass degradation by fungi, *in vitro *treatment of such biomass by lignin-degrading enzymes has a number of advantages, such as shorter incubation period without bacterial growth, reduced possibility of infection during large-scale microbe culture, lack of inhibitory effect of toxic byproducts (such as furfural) on bacterial or fungal mycelial growth, and improved reaction efficiency of bacterial enzymes at higher temperatures [1,5]. However, lack of commercially available, robust, and inexpensive enzymes is a major barrier for the widespread application of ligninolytic enzymes in various industrial sectors [5,6]. To avoid the high costs associated with enzyme purification procedures, one of the most promising ways to promote lignin biodegradation is to use crude enzymes [5-8].

Lignin is composed of chemically distinct subunits or monolignols (such as *p*-hydroxyphenyl, guaiacyl, and syringyl) whose abundance or proportions vary among plant species, and lignins of different plant origins require different proportions of laccase (E.C. 1.10.3.2), lignin peroxidase (LiP; E.C. 1.11.1.14) or manganese peroxidase, (MnP; E.C. 1.11.1.13) to promote the efficiency of degradation [2,3,9,10]. Laccase is the most preferred ligninase enzyme [11], but can directly oxidize only phenolic lignin units [12-14], which usually comprise less than 10% of the total polymer content of natural lignin [1,12]. By contrast, LiP is the most effective oxidizer ligninase known to date, and is capable of catalyzing the oxidation of phenolic or non-phenolic compounds, aromatic amines, aromatic ethers, and polycyclic aromatic hydrocarbons [13,14]. Thus, a laccase-LiP complex can be expected to be more efficient at lignin degradation than either of the two ligninases alone, owing to their potential synergism [5,6]. Further, laccases of fungal origin have low activity and stability at alkaline pH range or at high temperature, whereas laccases of bacterial origin have higher activity and stability under the same conditions [10,15]. One of the most studied aerobic cellulolytic bacteria species, *Streptomyces*, can produce ligninase with promising application potential under extreme conditions of high temperatures or high pH ranges [5,10,15].

Generally, strain selection or improvement by genetic modification, optimization of medium formulation, and fermentation conditions are the main options to improve enzyme activity and reduce production cost [5,6,10,15]. Compared with the high cost and uncertain results of genetic engineering, optimization of the medium formulation (including medium ingredients or composition, nutrient limitation, removal of potential toxic inducers) and culture conditions is a more dependable method in regulating the production of ligninolytic enzyme [5,16]. Carbon and nitrogen are the two most important components of the nutritional medium for any fermentation process [17,18], thus modifying or altering either or both in the medium should provide a marked improvement in the production of extracellular ligninolytic enzymes [10,18].

Currently, the most efficient method for large-scale fermentation is submerged fermentation (SmF; that is, an agitated liquid culture) [10]. Traditionally, production of ligninolytic enzyme by SmF usually has a higher production cost because the liquid media usually contains some relatively expensive constituents such as glucose, ammonium tartrate, nitriloacetic acid, MgSO_4_, NaCl, FeSO_4_, CoCl_2_, and ZnSO_4 _[19]. To decrease production cost, a potential solution is to adopt less expensive natural substrates as opposed to a chemically defined medium [20]. Coffee pulp, wheat bran, and yeast extract have been shown to be excellent substrates (nitrogen sources) for *Streptomyces psammoticus *to produce laccase by either SmF [21] or solid-state fermentation (SSF) [7]. Urea and sawdust have been used as nitrogen and carbon sources for laccase production from *Pycnoporus sanguineus *by SSF [22] and *Lentinula edodes *by SmF [23], respectively.

In this study, we aimed to produce a high activity laccase-LiP complex from a new strain of *Streptomyces cinnamomensis *by SmF using low cost liquid medium composed of yeast extract, coffee pulp, wheat bran, sawdust and urea.

## Results and discussion

### Principal component analysis

Laccase and LiP were simultaneously produced from *S. cinnamomensis *by SmF (Table 1).

**Table 1:** Simultaneous production of laccase and lignin peroxidase (LiP) from *Streptomyces cinnamomensis *by submerged fermentation

Run	Medium ingredients, g^a^					Activity, U/mL	
	
	Yeast extract	Coffee pulp	Wheat bran	Urea	Sawdust	Laccase^b^	LiP^b^
1	2.57	0.84	0.60	0.71	0.28	0.0004	0.0032
2	1.81	0.19	0.26	0.76	1.98	0.0023	0.0092
3	1.37	0.75	2.20	0.26	0.42	0.0002	0.0093
4	1.05	2.44	0.23	0.64	0.64	0.0060	0.0009
5	0.80	0.43	2.23	1.45	0.09	0.0062	0.0070
6	0.58	0.67	0.82	0.16	2.77	0.0007	0.0033
7	0.39	1.24	0.09	2.73	0.55	0.0039	0.1240
8	0.22	2.15	1.24	0.23	1.16	0.0041	0.0015
9	0.07	0.09	1.42	2.09	1.33	0.0065	0.0001
Mean ± SD	0.98 ± 0.81	0.98 ± 0.83	1.01 ± 0.82	1.00 ± 0.90	1.02 ± 0.88	0.0034 ± 0.0026	0.0176 ± 0.0400

^a^Basal medium for each run contained CaCO_3 _(0.02 g/L), MgSO_4 _(0.1 g/L) and trace element solution of 1%, with veratryl alcohol (sterilized using a 1 mmol/L filter) as inducer.

^b^Activity on the day 10 of submerged fermentation.

Following principal component analysis (PCA), four principal components of F_1_, F_2_, F_3_, F_4 _were calculated as shown in equations 1 to 4 below:

(1) $${\text{F}}_{1}=0.381+0.01778\times \left(\text{yeast}\;\text{extract}\right)+0.0792\times \left(\text{coffee}\;\text{pulp}\right)+0.462\times \left(\text{wheat}\;\text{bran}\right)-0.920\times \left(\text{sawdust}\right)$$

(2) $${\text{F}}_{2}=-0.578-0.0264\times \left(\text{yeast}\;\text{extract}\right)+1.046\times \left(\text{coffee}\;\text{pulp}\right)-0.344\times \left(\text{wheat}\;\text{bran}\right)-0.0692\times \left(\text{sawdust}\right)$$

(3) $${\text{F}}_{3}=-4.403+0.928\times \left(\text{yeast}\;\text{extract}\right)+1.040\times \left(\text{coffee}\;\text{pulp}\right)+1.387\times \left(\text{wheat}\;\text{bran}\right)+1.047\times \left(\text{sawdust}\right)$$

(4) $${\text{F}}_{4}=-0.459+1.073\times \left(\text{yeast}\;\text{extract}\right)-0.116\times \left(\text{coffee}\;\text{pulp}\right)-0.367\times \left(\text{wheat}\;\text{bran}\right)-0.110\times \left(\text{sawdust}\right)$$

The explained variances in F_1_, F_2_, F_3_, F_4 _were 28.649%, 25.610%, 24.721% and 20.661% respectively, and their cumulative percentage of variance reached 99.641%.

### Regression analysis and mathematical programming

Using linear regression, two second-degree polynomial regression models of laccase and LiP activities were constructed with four principal components (F_1 _to F_4_) as variables, as shown in equations 5 and 6 below:

(5) $$\text{Laccase}=0.01617+0.002399\times {\text{F}}_{2}-0.00337\times {\text{F}}_{3}-0.00179\times {\text{F}}_{4}-0.00258\times {\text{F}}_{1}^{2}-0.00410\times {\text{F}}_{2}^{2}-0.00460\times {\text{F}}_{3}^{2}-0.00313\times {\text{F}}_{4}^{2}\left(0.929,0.191\right)$$

(6) $$\text{LiP}=0.106+0.02944\times {\text{F}}_{2}-0.0290\times {\text{F}}_{3}+0.004978\times {\text{F}}_{4}-0.0144\times {\text{F}}_{1}^{2}-0.0470\times {\text{F}}_{2}^{2}-0.00910\times {\text{F}}_{3}^{2}-0.0294\times {\text{F}}_{4}^{2}\left(0.978,0.108\right)$$

Using unconstrained mathematical programming, the maximal enzyme activities and their corresponding solutions to principal components were calculated (Table 2).Based on the relationship between the principal component and the initial variables (yeast extract, coffee pulp, wheat bran and sawdust) presented in equations 2 to 4, the optimal dosages to the five medium ingredients corresponding to the maximum laccase or LiP activity could also be calculated (Table 2).

**Table 2:** Solutions to the expected maxima of laccase and lignin peroxidase (LiP) activities

	Laccase	LiP
Exp. max. activity	0.0174	0.1339
Component		
F_1_	-4.500 × 10^-8^	-2.700 × 10^-8^
F_2_	0.2926	0.3132
F_3_	-0.3663	-1.5934
F_4_	-0.2859	0.0847
Ingredient		
Yeast extract	0.62	0.74
Coffee pulp	1.15	0.97
Wheat bran	0.73	0.19
Sawdust	0.89	0.61
Ureaa	1.61	2.49

aUrea = 5 - (yeast extract) - (coffee pulp) - (wheat bran) - (sawdust).

### Design of verification experiment

For ligninolytic enzyme production, the types and concentrations of the carbon and nitrogen sources and the carbon:nitrogen ratio were shown to be key factors influencing extracellular laccase or peroxidase production from *Penicillium chrysogenum *[24], *Streptomyces *sp. F2621 and F6616 [16], *Streptomyces viridosporus *[25], *Streptomyces albus *[26], *Botryosphaeria rhodina *[27], *Pleurotus ostreatus *and *Pleurotus sajorcaju *[28], and *Streptomyces lavendulae *[5].

To verify the effect of the medium formulation on laccase and LiP activities and their activity ratio (laccase:LiP), three new medium formulations with different theoretical laccase:LiP activity ratios were designed (equations 7 to 9). For greater accuracy, the difference in variations (adjusted *R*^2^) and significance levels of the regression models of Equations. 5 and 6 were taken into account and quoted as parameters when designing the new medium formulations with different laccase:LiP activity ratios.

(7) $$0.1\;\text{laccase}\;\text{:}\;\text{1}\;\text{LiP = }\frac{0.1\times \text{Lac}\times \frac{0.929}{0.191}+1\times \text{LiP}\times \frac{0.978}{0.108}}{0.1\times \frac{0.929}{0.191}+1\times \frac{0.978}{0.108}}=0.1014+0.02806\times {\text{F}}_{2}-0.02769\times {\text{F}}_{3}+0.004633\times {\text{F}}_{4}-0.01380\times {\text{F}}_{1}^{2}-0.04481\times {\text{F}}_{2}^{2}-0.008870\times {\text{F}}_{3}^{2}-0.02806\times {\text{F}}_{4}^{2}$$

(8) $$1\;\text{laccase}\;\text{:}\;\text{1}\;\text{LiP = }\frac{1\times \text{Lac}\times \frac{0.929}{0.191}+1\times \text{LiP}\times \frac{0.978}{0.108}}{1\times \frac{0.929}{0.191}+1\times \frac{0.978}{0.108}}=0.07461+0.01999\times {\text{F}}_{2}-0.02004\times {\text{F}}_{3}+0.002613\times {\text{F}}_{4}-0.01027\times {\text{F}}_{1}^{2}-0.03201\times {\text{F}}_{2}^{2}-0.007530\times {\text{F}}_{3}^{2}-0.02022\times {\text{F}}_{4}^{2}$$

(9) $$2\;\text{laccase}\;\text{:}\;\text{1}\;\text{LiP = }\frac{2\times \text{Lac}\times \frac{0.929}{0.191}+1\times \text{LiP}\times \frac{0.978}{0.108}}{2\times \frac{0.929}{0.191}+1\times \frac{0.978}{0.108}}=0.05948+0.01544\times {\text{F}}_{2}-0.01573\times {\text{F}}_{3}+0.001473\times {\text{F}}_{4}-0.008280\times {\text{F}}_{1}^{2}-0.02478\times {\text{F}}_{2}^{2}-0.006770\times {\text{F}}_{3}^{2}-0.01549\times {\text{F}}_{4}^{2}$$

After unconstrained programming, the theoretical solutions to these three equations were calculated (Table 3).

**Table 3:** Theoretical solutions to medium formulations designed to produce laccase-lignin peroxidase (LiP) complexes with different laccase:LiP activity ratios

	Designed laccase:LiP ratio		
	0.1 laccase: 1 LiP^a^	1 laccase: 1 LiP^b^	2 laccase: 1 LiP^c^
Component			
F_1_	-7.800 × 10^-8^	-5.500 × 10^-8^	-5.400 × 10^-8^
F_2_	0.3131	0.3123	0.3115
F_3_	-1.5609	-1.3307	-1.1617
F_4_	0.0826	0.0646	0.0466
Ingredient			
Yeast extract	0.74	0.76	0.77
Coffee pulp	0.98	1.01	1.03
Wheat bran	0.2	0.28	0.35
Sawdust	0.61	0.66	0.69
Urea^d^	2.47	2.29	2.16

^a^Corresponding to max (0.1 × laccase + 1 × LiP) = 0.1276.

^b^Corresponding to max (1 × laccase + 1 × LiP) = 0.0911.

^c^Corresponding to max (2 × laccase + 1 × LiP) = 0.0711.

^d^Urea = 5 - (yeast extract) - (coffee pulp) - (wheat bran) - (sawdust).

### Result of verification

For the verification experiment using SmF culture at 25°C with shaking at 150 rpm (Table 4), on the 14th day of SmF with the optimal medium (w/v) of yeast extract 1.24%, coffee pulp 2.3%, wheat bran 1.46%, sawdust 1.78% and urea 3.22% (from Eqn 5) (Table 2; Table 4), laccase from *S. cinnamomensis *reached a peak of 0.0175 U/mL, which was approximately 5.1 times activity of the initial average value of 0.0034 U/mL (Table 1) and a little higher than the expected peak of 0.0174 U/mL (Table 2). This validated that laccase activity could be controlled by medium nutrition, and the optimized medium formulation was reliable for laccase production.

**Table 4:** Designed medium formulation for the verification experiment^a ^by submerged fermentation of *Streptomyces cinnamomensis*

	Expected maximum activity	Yeast extract	Coffee pulp	Wheat bran	Sawdust	Urea	Total
Laccase	0.0174	0.62^b ^(1.24%)^g^	1.15^b ^(2.3%)	0.73^b ^(1.46%)	0.89^b ^(1.78%)	1.61^b ^(3.22%)	5 (10%)
LiP	0.1339	0.74^c ^(1.48%)	0.97^c ^(1.94%)	0.19^c ^(0.38%)	0.61^c ^(1.22%)	2.49^c ^(4.98%)	5 (10%)

Designed laccase:LiP ratio							

0.1 laccase: 1 LiP	-	0.74^d^	0.98^d^	0.20^d^	0.61^d^	2.47^d^	5
1 laccase: 1 LiP	-	0.76^e^	1.01^e^	0.28^e^	0.66^e^	2.29^e^	5
2 laccase: 1 LiP	-	0.77^f^	1.03^f^	0.35^f^	0.69^f^	2.16^f^	5

^a^See Methods section for details.

^b^Solutions to max (laccase) in Table 2.

^c^Solutions to max (LiP) in Table 2.

^d^Solutions to max (0.1 × laccase + 1 × lLiP) in Table 3.

^e^Solutions to max (1 × llaccase + 1 × lLiP) in Table 3.

^f^Solutions to max (2 × llaccase + 1 × lLiP) in Table 3.

^g^1.24% = (0.62 g yeast extract)/(50 mL liquid medium) × 100%, the remaining percentage data in this table was calculated similarly.

After 7 days of SmF culture using the optimal liquid medium (w/v) of yeast extract 1.48%, coffee pulp 1.94%, wheat bran 0.38%, sawdust 1.22% and urea 4.98% (Eqn 6; Table 2; Table 4), LiP from *S. cinnamomensis *reached a peak of 0.1450 U/mL, which was approximately 8.2 times activity of the initial average value of 0.0176 U/mL (Table 1) and higher than the expected peak of 0.1339 U/mL (Table 2). This confirmed that the LiP activity could also be controlled by medium nutrition, and the optimized medium formulation was reliable for LiP production.

After 11 days of SmF culture using the three new medium formulations designed to produce laccase-LiP complexes with theoretical activity ratios of 0.0537 to 0.1 (Eqn 7), 0.537 to 1 (Eqn 8) and 1.074 to 2 (Eqn 9) (Table 3; Table 4), the measured activity ratios of laccase:LiP from *S. cinnamomensis *reached 0.1, 0.41 and 1.7 respectively (Table 5). The three activity ratios of laccase:LiP had nearly reached the designed levels of 0.05 to 0.1, 0.54 to 1 and 1.07 to 2 by day 11 of SmF fermentation, suggesting that the activity ratio between different enzymes produced simultaneously from one strain had the potential to be directly controlled by medium formulation. In addition, when the activity ratio of laccase:LiP reached the expected levels on day 11 of fermentation, the laccase or LiP activity also reached peaks (Table 5) on days 10,11 or 12, implying that the accordance of measured and theoretical laccase:LiP activity ratio was a true result as designed.

**Table 5:** Measured activity ratios of laccase-LiP complexes produced from *Streptomyces cinnamomensis *by submerged fermentation with optimized medium formulation

Day	Treatment								
	
	0.1 laccase/1 LiP			1 laccase/1 LiP			2 laccase/1 LiP		
	
	Laccase^a^	LiP^a^	Laccase: LiP	Laccase^a^	LiP^a^	Laccase: LiP	Laccase^a^	LiP^a^	Laccase: LiP
6	0.0017	0.0016	1.1	0.0010	0.0058	0.17	0.0006	0.0035	0.2
7	0.0010	0.0052	0.19	0.0006	0.0077	0.1	0.0007	0.0020	0.4
8	0.0009	0.0069	0.1	0.0003	0.0009	0.3	0.0021	0.0044	0.48
9	0.0019	0.0037	0.51	0.0004	0.0073	0.05	0.0008	0.0002	4
10	0.0005	0.0032	0.2	0.0006	0.0056	0.1	0.0015	0.0074^b^	0.20
11	0.0007	0.0075	0.1^c^	0.0019	0.0046	0.41^c^	0.0045^b^	0.0026	1.7^c^
12	0.0002	0.0114^b^	0.02	0.0027^b^	0.0096^b^	0.28	0.0041	0.0040	1.0
13	0.0009	0.0055	0.2	0.0030	0.0058	0.52	0.0015	0.0025	0.60
Range^d^	0.0537:1^e ^to 0.1:1			0.537:1^f ^to 1:1			1.074:1^g ^to 2:1		

^a^U/mL

^b^Activity peak of each enzyme produced using the designed medium.

^c^Measured activity ratios of laccase/LiP produced using the designed medium.

^d^Theoretical scope of laccase:LiP.

^e^(0.100 × 0.929/0.191)/(1 × 0.978/0.108).

^f^(1 × 0.929/0.191)/(1 × 0.978/0.108).

^g^(2 × 0.929/0.191)/(1 × 0.978/0.108).

## Conclusions

Compared with strain improvement by genetic engineering [2], optimization of medium formulation is a low-cost way to control enzyme activities and their ratio. By adjusting medium formulation, we produced a crude laccase-LiP complex with designed laccase:LiP activity ratio specially for the degradation of species-specific plant lignins with fixed proportion of phenolic units to non-phenolic compounds. Using such specially designed laccase-LiP complexes, the degradation of lignin and lignocellulose biomass should be improved. By improving the biodegradation of abundant and renewable lignocellulosic biomass, laccase-LiP complex with specially designed laccase:LiP activity ratio will be beneficial to industrial processes.

## Methods

### Materials

The *S. cinnamomensis *strain used was obtained from BOKU University of Natural Resources and Applied Life Sciences (Vienna, Austria). Wheat bran and sawdust were purchased, and coffee pulp was obtained from a common coffee machine in the Division of Food Biotechnolgy, Department of Food Sciences and Technology, BOKU - University of Natural Resources and Applied Life Sciences , Austria.

### Inoculum preparation

To prepare the inoculum, a growth medium composed of 15 g/L malt extract, 15 g/L yeast extract, 0.5 g/L (NH_4_)_2_SO_4_, 0.1 g/L CaCO_3_, 0.5 g/L MgSO_4_, and 0.5% (v/v) trace element solution (1% FeSO_4_, 0.09% ZnSO_4_, 0.02% MnSO_4 _(w/v) and distilled water) in 250 mL baffled flasks was autoclaved at 120°C for 20 minutes, then inoculated with *S. cinnamomensis* slant. The flasks were cultured at 25°C with shaking at 130 rpm for 3 days until spore concentration reached 1.5 × 10^7 ^colony-forming units (CFU)/cm^3^.

### Experimental design and fermentation

The medium for laccase and LiP production from *S. cinnamomensis *by SmF (Table 1) was prepared in Erlenmeyer flasks with veratryl alcohol as inducer [5,29]. After inoculation with 10% (7.5 mL v/v) of bacterial cell suspension containing 1.5 × 10^7 ^CFU/mL, 75 mL medium from each run was cultured at were cultured at 25°C with shaking at 120 to 130 rpm.

### Laccase activity assay

The laccase activity was determined by oxidation of 2,2'-azino-*bis*(3-ethylthiazoline-6-sulfonate) (ABTS, Sigma Chemical Co., St Louis, MO, USA) [5], with 1 U defined as the amount of laccase oxidizing 1 μmol of ABTS per minute [30].

### Lignin peroxidase activity assay

LiP activity was determined spectrophotometrically [5], with 1U of LiP activity defined as 1 μmol of veratryl alcohol oxidized in 1 minute [5,19].

### Spore concentration assay

Spore concentration was determined by measuring the absorbance at 650 nm [5]. The control had the same medium, but without inoculation.

### Data analysis

To fully analyze the possible interaction among the medium ingredients, a formulation design based on uniform design U_9_(3^9^) was adopted to optimize the medium formulation with five ingredients (Table 1). Corresponding to this formulation design, a second-degree polynomial regression model (Eqn 10) was constructed, with enzyme activity as the dependent variable [31]. After PCA, four principal components (F_1_, F_2_, F_3_, F_4_) corresponding to four ingredients (yeast extract, coffee pulp, wheat bran, sawdust) were constructed. Using unconstrained mathematical programming, the optimal solutions to F_1_, F_2_, F_3_, F_4 _were calculated when the dependents of enzyme activities reached maximum, then the optimal dosages of the four ingredients (yeast extract, coffee pulp, wheat bran, sawdust) could be calculated, and finally the optimal medium formulation could be fully calculated because the sum of five ingredients in each run was 5, hence:

urea=5-(yeastextract)-(coffeepulp)-(wheatbran)-(sawdust).

The enzyme activity model could be constructed as shown by Eqn 10:

(10) $$y=\beta_{0}+\beta_{1}\times {\text{F}}_{1}+\beta_{2}\times {\text{F}}_{2}+\beta_{3}\times {\text{F}}_{3}+\beta_{4}+{\text{F}}_{4}+\beta_{11}\times {\text{F}}_{1}^{2}+\beta_{22}\times {\text{F}}_{2}^{2}+\beta_{33}\times {\text{F}}_{3}^{2}+\beta_{44}\times {\text{F}}_{4}^{2}\;\left(R^{2},\;\text{significance}\right)$$

where *y *represents laccase or LiP activity; F_1_, F_2_, F_3 _and F_4 _were the principal components corresponding to the four ingredients of yeast extract, coffee pulp, wheat bran and sawdust in culture medium; β_i _was the partial regression coefficient with β_0_, β_1_, β_2_, β_3 _being the linear terms and β_11_, β_22_, β_33_, β_44 _being the quadratic terms. *R*^2 ^indicated to what extent of variability in the response could be explained by statistical model, and significance indicated the aptness of statistical model.

## List of abbreviations

CFU: colony-forming unit; LiP: lignin peroxidase; PCA: principal component analysis; SmF: submerged(/liquid) state fermentation; SSF: solid-state(/substrate) fermentation.

## Competing interests

The authors declare that they have no competing interests.

## Authors' contributions

DJ performed the literature retrieval, experimental design, experimental operation and data acquisition, data analysis and interpretation, and drafted the manuscript and submission. JW provided technical suggestions, and participated in the manuscript preparation and submission. Both authors read and approved the final manuscript.
